# Supplementary material for: Memory, Emotion, and Quality of Life in Patients with Long COVID-19
Source: Brain Sci. 2023 Dec 1;13(12):1670. doi: 10.3390/brainsci13121670 (PMC10742100; doi:10.3390/brainsci13121670)
Supplement: Supplementary file 1 [file brainsci-13-01670-s001.zip › brainsci-2721984-SI.pdf]

**Supplementary table 1 (*Table S1*).** Proof of normality.

| | | | |
|---|---|---|---|
| *Sociodemographic variables* | | | |
| | | Experimental (Kolmogorov-Smirnov) | Control (Shapiro-Wilk) |
| Age | | 0.080. p=0.200 | 0.970; p=0.591 |
| Sex | | 0.461; p=0.000** | 0.547; p=0.000** |
| Marital status | | 0.390; p=0.000** | 0.720; p=0.000** |
| Educational level | | 0.241; p=0.000** | 0.845; p=0.000** |
| Employement | | 0.266; p=0.000** | 0.897; p=0.000** |
| *Cognitive variables* | | | |
| *Montreal Cognitive Assessment (MOCA)* | | | |
| | | Experimental (Kolmogorov-Smirnov) | Control (Shapiro-Wilk) |
| Visuospatial | | 0.338. p=0.000** | 0544; p=0.000** |
| Identification | | 0.536; p=0.000** | |
| Attention | | 0.273; p=0.000** | 0.642; p=0.000** |
| Language | | 0.472; p=0.000** | 0.324; p=0.000** |
| Abstraction | | 0.536; p=0.000** | 0.324; p=0.000** |
| Memory | | 0.160; p=0.001* | 0.867; p=0.006* |
| Orientation | | 0.532; p=0.000** | |
| Total | | 0.182; p=0.000** | 0.936; p=0.150 |
| *List of words* | | | |
| | | Experimental (Kolmogorov-Smirnov) | Control (Shapiro-Wilk) |
| Immediate memory | | 0.069; p=0.200 | 0.976; p=0.830 |
| Delayed memory | | 0.155; p=0.002* | 0.916; p=0.054 |
| Recognition | | 0.320; p=0.000** | 0.662; p=0.000** |
| *Rey-Osterrieth Complex Figure Test* | | | |
| | | Experimental (Kolmogorov-Smirnov) | Control (Shapiro-Wilk) |
| Copy | Time | 0.104; p=0.200 | 0.932; p=0.139 |
| | Score | 0.154; p=0.003* | 0.780; p=0.000** |
| Immediate memory | Time | 0.210; p=0.000** | 0.975; p=0.796 |
| | Score | 0.114; p=0.080* | 0.966; p=0.594 |
| Delayed memory | Time | 0.147; p=0.005* | 0.965; p=0.562 |
| | Score | 0.132; p=0.020* | 0.955; p=0.374 |
| *Digit Span Task* | | | |
| | | Experimental (Kolmogorov-Smirnov) | Control (Shapiro-Wilk) |
| Digit Span Forward Test | | 0.207; p=0.000** | 0.931; p=0.116 |

| | | | |
|---|---|---|---|
| Digit Span Backward Test | | 0.216; p=0.000** | 0.890; p=0.016* |
| Total Forward Digits | | 0.169; p=0.001* | 0.946; p=0.243 |
| Total Backward Digits | | 0.191; p=0.000** | 0.958; p=0.421 |
| *Trail Making Test (TMT)* | | | |
| | | Experimental (Kolmogorov-Smirnov) | Control (Shapiro-Wilk) |
| | Trials | 0.539; p=0.000** | |
| TMTa | Errors | 0.532; p=0.000** | |
| | Time | 0.132; p=0.020* | 0.736; p=0.000** |
| | Trials | 0.522; p=0.000** | 0.356; p=0.000** |
| TMTb | Errors | 0.484; p=0.000** | 0.469; p=0.000** |
| | Time | 0.218; p=0.000** | 0.591; p=0.000** |
| *Cancellation Task* | | | |
| | | Experimental (Kolmogorov-Smirnov) | Control (Shapiro-Wilk) |
| TOT Effectiveness | | 0.108; p=0.172 | 0.885; p=0.013* |
| CON Concentration index | | 0.107; p=0.178 | 0.951; p=0.302 |
| TR Total of responses | | 0.105; p=0.200* | 0.931; p=0.000** |
| TA Total of trials | | 0.113; p=0.000* | 0.950; p=0.294 |
| Comissions | | 0.530; p=0.000** | 0.324; p=0.000** |
| Omissions | | 0.193; p=0.000** | 0.894; p=0.000** |
| *Rivermead Behavioural Memory Test (RMBT)* | | | |
| | | Experimental (Kolmogorov-Smirnov) | Control (Shapiro-Wilk) |
| Recalling the date | | 0.346; p=0.000** | 0.536; p=0.000** |
| Recalling of object | | 0.496; p=0.000** | 0.184; p=0.000** |
| Recalling of place | | 0.504; p=0.000** | |
| *Digit Symbol Coding* | | | |
| | | Experimental (Kolmogorov-Smirnov) | Control (Shapiro-Wilk) |
| Score | | 0.067; p=0.200 | 0.873; p=0.007* |
| Incidental memory | | 0.142; p=0.008* | 0.836; p=0.002* |
| *Boston Vocabulary Test* | | | |
| | | Experimental (Kolmogorov-Smirnov) | Control (Shapiro-Wilk) |
| Spontaneous answers | | 0.162; p=0.001** | 0.897; p=0.022** |
| Semantic key | | 0.432; p=< .001** | 0.592; p=< .001** |
| Phonological key | | 0.199; p=< .001** | 0.888; p=0.014** |
| *Verbal Fluency Task* | | | |
| | | Experimental (Kolmogorov-Smirnov) | Control (Shapiro-Wilk) |

| | | |
|---|---|---|
| Words with P- and S- (Spanish language) | 0.072; p=0.200* | 0.990; p=0.997* |
| Animals | 0.65; p=0.200* | 0.927; p=0.095* |
| *Psychological Variables* | | |
| | Experimental (Kolmogorov-Smirnov) | Control (Shapiro-Wilk) |
| Beck Depression Inventory (BDI-2) | 0.136; p=0.014** | 0.838; p=0.002** |
| STAI-Stait | 0.130; p=0.023** | 0.934; p=0.133 |
| STAI-Trait | 0.120; p=0.051 | 0.944; p=0.218 |
| Oviedo Sleep Questionnaire | 0.069; p=0.200 | 0.904; p=0.031* |
| Modified Fatigue Impact Scale (MFIS) | 0.105; p=0.200 | 0.897; p=0.022* |
| Memory Failures of Everyday (MFE) | 0.142; p=0.001* | 0.824; p=0.001* |
| SF-12 Health Survey | 0.108; p=0.177 | 0.936; p=0.148 |

*p<0.05; **p<0.001. lower limit of true significance
